# Inhabiting the Body(ies) in Female Soccer Players: The Protective Role of Positive Body Image

**DOI:** 10.3389/fpsyg.2021.718836

**Published:** 2021-09-24

**Authors:** Débora Godoy-Izquierdo, Isabel Díaz

**Affiliations:** ^1^Department of Personality, Evaluation and Psychological Treatment, Faculty of Psychology, University of Granada, Granada, Spain; ^2^Women's and Gender Studies Research Institute, University of Granada, Granada, Spain; ^3^Gimbernat-Cantabria Research Unit (Servicio Universitario de Investigación Gimbernat-Cantabria), University School Gimbernat-Cantabria, University of Cantabria, Torrelavega, Spain

**Keywords:** body image, body positivity, eating pathology, women, gender, soccer (football), feminist sport psychology

## Abstract

In a social and sports world dominated by weight-centred beliefs and highly exigent and gendered athletic and beauty body standards, the vulnerability for eating pathology, i.e., disordered eating and eating disorders (EDs), is elevated among women athletes. The aim of this study was to explore body image facets and ED risk among female athletes in masculinised sports such as soccer. Forty-five federated amateur female soccer players from Spain participated in this study, voluntarily complying with an extensive evaluation protocol on attitudes towards body and appearance and eating practises. The participants overall reported self-representations of their bodies that corresponded to their reality as athletes, but their body ideals were also more demanding in terms of low fat and muscularity, in association with the functionality of their body and the physical demands of their athletic activity. Despite having a fairly high positive body image and body satisfaction, they also expressed negative attitudes towards their bodies. Around 2 out of 10 players were at risk of suffering from an ED. Players with negative attitudes towards their bodies had an odd 12 times likely to develop an ED compared to those with lower self-devaluation, after adjusting for BMI and body perceptions (OR = 12.3, *p* < 0.01). On the contrary, players who appreciate their bodies and hold a positive body image had an odd 83% lower to suffer from eating pathology, after adjusting for BMI and body satisfaction (OR = 0.17, *p* < 0.05). Our findings support the healthy and protective role of positive body image in sports contexts. Body attitudes should be addressed in preventive and therapeutic efforts for reducing the prevalence of EDs in women's sports, within both a “negative” and a “positive” paradigm of body image.

## Introduction

Female athletes in general have better body perceptions and higher body satisfaction than non-athletes, and a poorer body image is associated with lower sport commitment and performance (Voelker and Reel, 2018; Sabiston et al., 2019). Some scholars have emphasised that sport participation may protect athletes from body image and eating dysfunctions, but also that special attention should be devoted to issues affecting female athletes, e.g., internalisation of beauty ideals emphasising appearing both thin-feminine and athletic, pressures focused on weight and appearance added to pressures for excellence, participation in traditionally feminine vs. masculine sports and increased sexual objectification in the media and society (Varnes et al., 2013). These phenomena may explain the increased risk for eating pathology among sportswomen compared to the general population (Bratland-Sanda and Sundgot-Borgen, 2013; de Bruin, 2017). A feminist framework is helpful for a deeper understanding of all these issues (Busanich and McGannon, 2010).

Sportswomen, in addition to the strict societal canons of feminine beauty, also experience specific body-focused pressures due to the sport they practise. Many female athletes claim to have positive body perceptions in sports contexts but feel concerned about their current and “ideal” body weight, size and shape and are dissatisfied with their body image, mainly due to the perceived tensions between the performative body –which emphasises strength, size and functionality– and the hegemonic ideals of the female body –which privilege thin, curved, toned, heterosexual bodies– (Kong and Harris, 2014; Liechty et al., 2015; Bennett et al., 2016; Lunde and Gattario, 2017; Cosh et al., 2019; Devonport et al., 2019). Vicky Krane called “paradox” to the continuous negotiation that athletes have to make regarding femininity and athleticism (Krane et al., 2004). As she stated, sportswomen live simultaneously in two cultures, being marginalised in both: the sports culture, that is inherently masculine, and the larger social culture, where femininity is celebrated for women. For female athletes, being feminine contrasts with being athletic. Within hegemonic femininity, the athlete is (self)perceived as “other” by their physicality; as women, they are perceived as different from “normal” women. Even so, they are also proud of their strong and powerful bodies and express feelings of empowerment that generalised beyond the sport context. Thus, a contextual body image perspective (i.e., sport context, daily life context) is needed to understand how the athletes self-perceive and value their bodies and the transiency of their bodily experiences (de Bruin et al., 2011).

In addition, some sports have been identified as associated with a higher risk for problematic attitudes and behaviours related to body image and thus for disordered eating and eating disorders (EDs), particularly those in which body and weight are more relevant for sport performance, namely weight-sensitive or leanness sports (Smolak et al., 2000; Sundgot-Borgen et al., 2013). Research on body image and eating pathology is extensive in aesthetic sports, in which a slim, small and slender body is valued and required not only because it is stereotypically associated with better performance, but also because athletes' bodies are noticeable (e.g., outfits) and appearance is a component in the evaluation of athletes' performance. Among sportswomen in these modalities, a high risk of eating pathology has been reported (e.g., Kong and Harris, 2014; Neves et al., 2017; de Bruin and Oudejans, 2018; Kantanista et al., 2018; Teixidor-Batlle et al., 2019; Mancine et al., 2020). There is also abundant research on body image in endurance, weight-classes and power sports (e.g., Folscher et al., 2015; Rodríguez et al., 2015; de Bruin and Oudejans, 2018; Kantanista et al., 2018; Mathisen and Sundgot-Borgen, 2019; Stoyel et al., 2021). In recent years, interest has grown on these issues in other sports such as ball sports, in which, with some exceptions such as volleyball, it has been traditionally assumed that there is *a low pressure* on the body, and consequently low risk for disordered eating (Díaz et al., 2018). However, contrary to expectations, research in ball sports such as soccer is demonstrating that concerns about body weight and appearance are also high, and pressures regarding size, shape and weight are also elevated (e.g., Liechty et al., 2015; Lunde and Gattario, 2017; Kantanista et al., 2018; Godoy-Izquierdo et al., 2019a; Kampouri et al., 2019; Heradstveit et al., 2020). In the dual-pathway etiological model proposed by Petrie and Greenleaf (2012) and Stoyel et al. (2020), body dissatisfaction is a result of the internalisation of societal and sport-specific pressures, and considered a risk factor for eating pathology among athletes. Nevertheless, this model ignores the gendered nature of body image (Krane et al., 2001). In sports, bodily experiences of women and men differ, and men are not exposed to the same social and sport pressures regarding their body than women athletes (Wilson et al., 2016).

Furthermore, the pressures experienced by female athletes who play sports such as soccer are different to those of sportswomen in other sports. As each sport requires an optimal physique to conquer excellence, the bodies of soccer players diverge from those in other modalities. Within the current ideals of beauty and femininity, not all bodies and shapes are accepted for a female athlete. A large, muscular and strong body such as that of a female soccer player is particularly sanctioned, because it is “virile,” a masculinised body that is distant from the docile, fragile and beautiful body valued for a woman, and a female athlete. Yet currently a toned, firm body is also (self)valued for girls and women as long as it is thin and feminine, with beauty patterns changing over time from the glorification of extreme thinness to demanding slim and athletic bodies in women (Kong and Harris, 2014), this is not the same as accepting bodies that are entering the domain of men and defying their power. Thus, female athletes in power-based sports, where the focus tends to be on more masculine qualities such as strength and muscularity (e.g., soccer, field hockey, rugby), have reported feeling that their bodies are under constant scrutiny as they are permanently tasked with the responsibility of promoting/conforming to a brand image that emphasises femininity (Perry et al., 2021). For all this, women competing in masculinised sports face a double pressure regarding their weight and appearance: on the one hand, the eternal battle between the social or aesthetic body and the athletic or instrumental body and, on the other, an added continuous struggle between the feminine athletic body and the masculinised athletic body by the sport they compete in, giving place to new phenomena such as the stigma of being called “butch” and “lesbian” as an insult due to their appearance (Liechty et al., 2015; Bennett et al., 2016; Lunde and Gattario, 2017; Cosh et al., 2019; Perry et al., 2021).

Therefore, female athletes in sports such as soccer navigate two opposed realities in their day-to-day lives, one in which their bodies are perceived as too large and muscular for the sociocultural standards of female beauty (in their daily life, with their feminine identity being affected), and another one in which their bodies are not cultivated or powerful enough and could be even more big and muscular for performance enhancement (in their athletic life, with their athletic identity being affected). As others have claimed (Lunde and Gattario, 2017), female athletes are in the intersection between the culture within the sport –emphasising physical performance– and the culture outside the sport –emphasising physical appearance–, articulating a permanent internal dialogue and witnessing a conflict for not fully fitting into either of these two scenarios. In fact, these athletes have reported that they feel satisfaction and pride in their physique, but concerns regarding being (necessarily) excessive muscled, and that they tend to value their bodies differently depending on whether they are in a sports or a social context (e.g., they feel satisfied with their bodies in sports settings and when surrounded by teammates, but dissatisfied in social settings due to comparisons with the bodies of their non-athlete peers) (Liechty et al., 2015; Bennett et al., 2016; Lunde and Gattario, 2017; Cosh et al., 2019; Devonport et al., 2019). Moreover, it has been found that female athletes are often judged on their appearance rather than their performance and most express a desire to change their physique for aesthetic reasons rather than health or functionality (Bennett et al., 2016; Lunde and Gattario, 2017; Cosh et al., 2019). In addition, attitudes and behaviours such as eating large amounts of food due to physiological needs can cause criticism that affects athletes (Lunde and Gattario, 2017); these criticisms are beyond the myth that “the lower the weight and the fat percentage, the better the performance,” entailing an expectation of what is appropriate for women.

Feminist scholars have critically analysed and discussed female athletes' body image and bodily experiences to interpret them within the framework of the hegemonic patriarchy and to confront them as a form of discrimination, subordination, exploitation and invisibility towards women in the fields, courts, tracks and competitions. These researchers propose sport activity as a double-edged sword for women which can both promote and harm body satisfaction, and warn about the paradox of the female athlete, which argues that the strong and athletic body does not align with the feminine ideal (Liechty et al., 2015; Lunde and Gattario, 2017; Eke et al., 2020). A further assumption is that gender is performed (Butler, 1990) and that femininity acting in the case of athletes is performed both in everyday and sports life spheres (Devonport et al., 2019). As a consequence, there is a permanent transiency of self-perceptions, bodily satisfaction and personal identity and agency across both contexts (Bennett et al., 2016; Lunde and Gattario, 2017; Devonport et al., 2019). Through their sports participation, female athletes, and soccer players in particular, can experience an improvement in their body image as a result of body changes and the appreciation of their bodies' functionality over their appearance, this modifying their beliefs and values of beauty. They can thus value and celebrate “other” bodies, based on the recognition of diversity. Sport can provide an opportunity to resist and challenge the hegemonic discourses on the body and the various forms of control and constraint imposed on their bodies, to negotiate the contrary demands of femininity and athleticism, to inspire self-constructions that claim other corporealities or “somebodiness” and to subvert the dominant discourses on (hetero)sexual attractiveness. But sportswomen have to constantly articulate their body image as well as their personal and gender identity inside and outside the field (Wilinski, 2012). Many athletes, particularly those who compete in masculine sports such as soccer, find it very difficult to adjust to non-dominant forms of corporality and engage in surveillance and management practises (e.g., dieting, clothing, make-up) that serve to comply and thus perpetuate the sociocultural values of the feminine, assuming a heterosexual(ised) body and legitimising athletic participation through an emphasis on hyperfemininity (Fisher and Dennehy, 2015; Heinecken, 2015; Bennett et al., 2016; Lunde and Gattario, 2017; Cosh et al., 2019; Devonport et al., 2019; Sanders, 2020).

Alternative discourses on females' body image in sports are necessary to help athletes to gain empowerment regarding their corporeality and embodiment experiences. Recently, the field of positive body image is flourishing (Tylka and Wood-Barcalow, 2015a; Tylka, 2018). Based on seminal contributions in the study of negative and positive facets of body image along with feminist influences such as the embodiment theory, positive body image is proposed as a multidimensional, holistic and protective construct distinct from negative body image, which is mostly raised from and focused on pathology. It is not a continuum similar to negative self-representations, nor the opposite endpoint in a continuum of body image. It is composed of several facets involving much more than body satisfaction or appearance evaluation, including body appreciation, body acceptance and love, conceptualising beauty broadly, adaptive investment in appearance, inner positivity and interpreting information in a body-protective manner. Positive body image has been defined as “an overarching love and respect for the body that allows individuals to (a) appreciate the unique beauty of their body and the functions that it performs for them; (b) accept and even admire their body, including those aspects that are inconsistent with idealised images; (c) feel beautiful, comfortable, confident, and happy with their body, which is often reflected as an outer radiance, or a “glow”; (d) emphasise their body's assets rather than dwell on their imperfections; and (e) have a mindful connection with their body's needs; and (f) interpret incoming information in a body-protective manner whereby most positive information is internalised and most negative information is rejected or reframed” (Wood-Barcalow et al., 2010, p. 112). As such, positive body image involves appreciating the unique body beauty and functionality, valuing body qualities beyond or regardless of actual physical appearance, feeling self-worth by highlighting body's assets while minimising perceived imperfections, defining beauty broadly and appreciating diverse appearances, having favourable opinions on and accepting one's own body despite incongruence with appearance ideals, filtering information (e.g., appearance commentaries, media ideals, external pressures) in a protective manner against unrealistic beauty ideals (e.g., contrary to internalisation, self-objectification and social comparison) and respecting and taking care of the body by attending to its needs and decreasing body-appearance investment (e.g., healthy behaviours). Individuals high in body positivity appreciate, respect, celebrate and honour their bodies, they are *connected* with their bodies in an appreciative and caring manner. As such, positive body image has unique associations with well-being, health, self-care and behaviour that are not accounted for solely by (lack of) negative body image. Yet positive body image operates similarly across a range of populations, it is also shaped by social identities. Thus, gender, age, culture, race, size, ability, sexual orientation, religion/spirituality and socioeconomic status (Tiggemann, 2015; Tylka and Wood-Barcalow, 2015a) allow body positivity to express uniquely in different contexts. Thus, to gain understanding on positive body image, additional research with an intersectional approach is paramount.

The nuances of positive body image in female athletes have been recently, yet still scarcely, studied, supporting its role in promoting positive sport experiences related to the body and eating attitudes and behaviours. For instance, it has been found that student athletes report higher body appreciation and higher functionality appreciation than non-athletes, and that female athletes report lower levels of positive body image than male athletes; moreover, positive body image has been related to sport-related variables such as sport confidence, positive subjective states and subjective sport performance (Soulliard et al., 2019). Among athlete girls, greater body appreciation has been found compared to non-athletes, irrespective of whether they were involved in weight-sensitive or non-weight-sensitive sports. Furthermore, body appreciation and body functionality are associated with lower disordered eating (Jankauskiene et al., 2020). Self-compassion—as closely related to body appreciation (Homan and Tylka, 2015; Andrew et al., 2016)—helps female athletes to recognise the uniqueness of sport contexts, promote compassionate body awareness and set realistic standards and expectations for themselves, feeling higher body appreciation in their sporting experiences and adhering to intuitive eating (Killham et al., 2015). Consequently, it has been proposed that positive body image can help female athletes to experience more positive embodiment experiences, greater health and well-being and lower risk for negative body image and disordered eating (Menzel and Levine, 2011).

The present study aims to explore the body image of female athletes in masculine sports such as soccer. In the literature reviewed, there are hardly any studies on Spanish soccer players, hence the need to further understand how female soccer players perceive their bodies, regulate their bodily self-values and negotiate their identities, and the influences of these issues on their body and appearance management behaviours. Specifically, we addressed players' body perceptions, their attitudes towards their weight and appearance, their body appreciation and their body satisfaction, within a “positive” and a “negative” body image paradigm for a more comprehensive understanding of body image facets. We further addressed the associations of body self-representations and attitudes with risk for disordered eating. Based on previous evidence, we expected to find that a noticeable percentage of the participants would show negative body perceptions, unfavourable attitudes towards their body and appearance and a high risk for eating pathology. Specifically, based on previous research with Spanish athletes (Teixidor-Batlle et al., 2021) and soccer players (Godoy-Izquierdo et al., 2019a; Petisco-Rodríguez et al., 2020), we expected to find that ~1 in 10 players exhibited high-risk beliefs, attitudes and behaviours related to body, food and weight. In addition, we expected that the participants would also report high body appreciation, and these positive self-perceptions would in turn diminish their risk for eating pathology.

## Materials and Methods

### Participants

A total of 45 Spanish federated non-elite female soccer players participated in this study. They voluntarily completed an extensive evaluation protocol on attitudes towards the body and appearance and eating practises as a part of a broader research on body image in female athletes. Their age ranged from 13 to 44 years (20.9 ± 7.5 years). These players belonged to national soccer clubs and teams competing at the two highest levels of amateur (non-professional) female soccer. Regarding their athletic history, there is great diversity in trajectories. The time competing in soccer ranges from 1 to 21 years, but many indicated practising/having practised and competing/having competed in other sports modalities and soccer specialties. Other sociodemographic and athletic data are displayed in Table 1. Interestingly, none indicated suffering from diagnosed disordered eating or EDs.

**Table 1:** Female soccer players' sociodemographic and athletic data.

		**%**
Nationality	Spanish	93.3
	Residing in Spain > 3 years	6.7
Education level	Primary school	6.7
	Secondary school	44.4
	Professional training	20
	University (graduate, postgraduate)	28.9
Work status	Student	75.6
	Employed	13.3
	Professional athlete	2.2
	Unemployed	8.9
Relationship status	Single	11.1
	Non-stable commitment	53.3
	Stable commitment	35.6
Athletic history (soccer)^a^	1–2 years	37.8
	3–5 years	37.8
	6–8 years	13.3
	9 years or more	11.1
Performance level^b^	1st National	22.2
	Regional Preferente	77.8
Playing position^c^	Goalkeepers	4.4
	Defenders/Centre-backs/Full-backs	24.4
	Midfielders/Half-backs	20.0
	Central/Inside forwards	13.3
	Wingers	37.8
Training volume: weekly frequency	1–2 days	44.4
	3–4 days	44.4
	5 days or more	11.1
Training volume: weekly duration	3 h or less	11.1
	4–6 h	48.9
	7 h or more	40
Previous experience in practising/	No	55.6
competing in other sports	Non-competitive practise	37.8
	Competitive practise	6.7
Current competition in other sports	No	77.8
	Yes	22.2
Athletic injury at the time of the study	No	100

a *When a participant indicated a stoppage in her athletic career, history was considered from the moment of restarting the activity (although this decision implies losing an important practise history, it was not easy many times to determine her previous practise time or the duration of the withdrawal period).*

b *Performance level in Spanish female soccer is categorised in: 1st Division (professional soccer) and 2nd Division (semi-professional soccer), both dependent on the Professional Soccer National League and competing at a national level; 1st National Female Soccer (amateur soccer), dependent on the Spanish Royal Soccer Federation and competing at an autonomic level; and Regional-Preferente, 1st Regional and 2nd Regional (amateur soccer), dependent on the Autonomic Federations and competing at a regional level.*

c *Playing positions were grouped according to the body required or expected for each position by the players' performance in each one; the specific nutritional requirements of each playing position were also considered (Randell et al., 2021)*.

### Measures

This study is part of a larger investigation on body image in women's sports. In the present study, measures were used to collect information on personal data (age, nationality and place of residence, with indication of time of residence, educational level, employment status and relationship status; all the participants self-identified themselves with female sex-gender) and sports data (team/club, non-competitive and competitive practise in soccer and other sports with indication of sport type, weekly training volume –time and frequency–, playing position and injuries at the time of the study with indication of type, time and severity), as well as self-reported weight and height (from which BMI was calculated as kg/m^2^; Santos et al., 2015), body image and attitudes and issues related to nutrition and weight management, including risk for EDs. Body image has been traditionally understood as a multidimensional construct that broadly incorporates the experiences and self-representations related to the weight, shape and appearance of the body and/or its parts, involving perceptual (i.e., weight and shape estimations), evaluative (i.e., attitudes, evaluations and judgments with a subjective or affective dimension, such as body satisfaction) and behavioural components (i.e., strategies to manage weight, control the body and transform the appearance, including eating, exercise, hiding/exposing, ornamentation, etc.) (Thompson et al., 1999; Cash and Pruzinsky, 2002; Grogan, 2017). All these facets of body image were evaluated: the perceptual, by means of body estimations; the evaluative-attitudinal, by means of body satisfaction and body attitudes; and the behavioural dimension, by means of disordered eating and eating symptoms. Body appreciation was also assessed as a core facet of positive body image.

Specifically, body perceptions were evaluated using silhouettes corresponding to different values of BMI and muscle tone, thus accounting simultaneously for the dimensions of weight/shape and composition/muscularity (Ramírez et al., 2015, 2018). For this, 15 silhouettes corresponding to women's bodies were presented, with silhouette number 1 being that of a body with a high degree of obesity and very low muscle tone, number 8 that of a very thin and very flabby body and number 15 that of a very muscular body with very low level of fat. The participants indicated which one of these silhouettes corresponded to their current body perceptions (perceived body image, PBI) and ideals (ideal body image, IBI). Figural rating scales or anatomical models have been widely used to evaluate representations of the body, usually with different shapes in terms of body size or weight and sometimes also including body structure or composition, and they have good psychometric properties as well as cross-cultural validity (Gardner and Brown, 2010).

Body satisfaction was assessed by a face-valid question about the degree of satisfaction with the current body and physical appearance (“How satisfied are you with your current body shape and weight?,” 1 = extremely dissatisfied, 7 = extremely satisfied), as well as through the PBI-IBI discrepancy, computed as the difference between PBI and IBI values (Ramírez et al., 2015, 2018). This type of indicators have been widely used to evaluate the subjective-evaluative component of body image and their psychometric properties have been widely established (Thompson, 2004).

To assess negative attitudes towards the body and appearance, the Ben-Tovim and Walker's Body Attitudes Questionnaire (BAQ) (Ben-Tovim and Walker, 1991) was used. Through 44 items (1 = completely disagree, 5 = completely agree), six dimensions of experiences related to the body are evaluated: (Absence of) Attractiveness (5 items); Salience of weight and shape (8 items); Body self-disparagement (8 items); Feelings of overall fatness (13 items); Feelings of fatness in the lower part of the body (4 items); and (Absence of) Strength/Fitness (6 items). Higher scores indicate more unfavourable or unhealthy attitudes. The BAQ psychometric properties in terms of reliability and convergent validity with other measures of attitudes towards body weight and appearance, drive for thinness, body dissatisfaction and eating pathology are satisfactory and it appropriately discriminates between females with and without eating pathology (Probst et al., 2008, 2009). Translation and backtranslation procedures were used for obtaining the Spanish version (available upon request). Cronbach's *alpha* in this study was 0.92 for the total scale.

Positive body image was assessed with the Body Appreciation Scale-2 (BAS-2) (Tylka and Wood-Barcalow, 2015b; Spanish version by Swami et al., 2017), which is the most used scale to evaluate components of the positive body image (Webb et al., 2015). This self-report assesses the three core facets of body image under the conception of positive body self-representations: (a) acceptance and favourable valuation of one's own body with its weight, shape, features and functions; (b) respect and attention to the needs of the body through the adoption of self-caring healthy behaviours; and (c) self-protection through rejecting the unrealistic bodily ideals presented in the media as the only model of beauty and resistance to the internalisation of these ideals. The 10 items on the BAS-2 are answered on a scale of 1 = never to 5 = always, and are averaged to obtain an overall body appreciation score. Higher scores reflect a greater appreciation of the body. The BAS-2 has been validated in the Spanish population (Swami et al., 2017) and adequate psychometric properties have been found, showing direct associations with healthy evaluations of appearance, body satisfaction and self-esteem and inverse with BMI. The BAS-2 has also been inversely associated with drive for thinness, body shame, body dissatisfaction, internalisation of the media appearance ideals, body surveillance and risk for EDs (Tylka and Wood-Barcalow, 2015b). The scale Cronbach's *alpha* was 0.96 in this study.

The risk for eating pathology was evaluated through a general screening questionnaire and a specific one for the sports population (Pope et al., 2015):

- Eating Attitudes Test (EAT-26) (Garner et al., 1982; Spanish version by Rivas et al., 2010). The EAT-26 assesses the risk for eating pathology through three dimensions evaluating attitudes towards weight and food, concerns about the body and eating and risky weight management practises: Dieting/eating restriction (13 items on food avoidance behaviours, eating practises and preoccupation with appearance), Bulimia and preoccupation with food (6 items referring to bulimic behaviours and concerns associated with eating) and Oral and external control (7 items on self-control of food intake and external pressures to eating). Responses on a 6-point Likert scale are recoded from 0 = never, rarely, sometimes to 3 = always. The EAT-26 has been widely used as a screening, not as a diagnostic, tool in non-clinical and clinical samples, also in the sports population (e.g., Teixidor-Batlle et al., 2021). In Spanish samples, it has been proposed that a score between 10 and 19 indicates moderate risk, and 19 or above indicates high risk (Rivas et al., 2010), thus lowering by 1 point the established cut-off point (Garner et al., 1982) (However, adopting the classical cut-off does not alter any of the findings). Cronbach's *alpha* was 0.92 for the total scale.
- Athlete's Eating Habits Questionnaire-Brief (CHAD-B) (Godoy-Izquierdo et al., 2017), revised, brief version of the Athlete's Eating Habits Questionnaire (CHAD) (Dosil et al., 2012) (available upon request). The original CHAD was specifically designed to assess risk for eating pathology in athletes and it has been used in both sporting and exercise contexts. It consists of 30 items in four factors: FI: Fear of gaining weight during resting periods and use of exercise for weight control, FII: Distress related to weight and body appearance due to attitudes and comments from significant others, FIII: Obsessive worries and concerns about food, diet and weight and FIV: Body image and body dissatisfaction. The athlete responds on a Likert-type scale from 1 = totally disagree to 6 = totally agree, with higher scores indicating higher risk. The psychometric properties of the original CHAD had been previously demonstrated (Dosil et al., 2012; Godoy-Izquierdo et al., 2019b). Content and psychometric analyses led its authors to develop the abbreviated 20-item version (FI: 4 items; FII: 5 items; FIII: 6 items; FIV: 5 items). Preliminary findings support the good psychometric properties of the CHAD-B (Godoy-Izquierdo et al., 2017, 2019b). A score of ≥60 points indicates high risk, and ≥66 is the cut-off point for determining very high risk (Godoy-Izquierdo et al., 2017). The scale Cronbach's *alpha* was 0.96 in this study.

### Procedure

As part of a research-intervention parent study, several national women's soccer clubs and teams contacted the authors requesting psychological advice for their players on issues related to eating behaviours, weight management and the prevention of eating pathologies. The contactands—mainly coaches—were unaware of the potential eating or body image issues of their players, nor were they particularly concerned about the participating players. In addition, their demand was not based in turn on demands from players. All the players in their teams were informed of this interest by the technical bodies of their teams or clubs and were offered the opportunity to voluntarily participate. Those who accepted were contacted by the researchers to provide them with detailed information on the objectives, components and contents of the research, as well as their rights and tasks as participants. Then, all of them signed an informed consent for participation. The underage players informed their parents/legal guardians and both the participants and the latter gave their consent.

In a second phase, they all participated in a baseline assessment. To do this, we previously created an online survey that included all the self-reports described in the Measures section, along with others whose information is not analysed in this paper. A link was then provided to the coaches of each team so that they could disseminate it among the players of their teams. The players accessed this survey in 2 specific weeks (01/27/2021–06/02/2021). The database was then downloaded and checked in detail for the detection and correction of possible errors or missing data. The final sample comprised all the participants who were initially contacted, once they reported their willingness to collaborate. Thus, no participant was excluded and there was no withdrawal during the study.

The study was conducted according to the guidelines of the Declaration of Helsinki and approved by the Institutional Ethics Committee of the first author's institution (Ref. 2230).

### Study Design and Data Analyses

This is an observational-descriptive study with a correlational, cross-sectional design. Descriptive analyses (mean and standard deviation, *n* and %) and non-parametric analyses of Spearman's *rho* correlations and Mann-Whitney's *U* pair comparisons were performed. In addition, in order to determine the Odds Ratio (OR) for eating pathology vulnerability considering the main study variables as predictors, a multivariate logistic regression with maximum likelihood method was conducted for each screening tool separately (i.e., EAT-26 and CHAD-B). The predicted variable was dichotomised in 0 = no risk, 1 = risk considering the participants' scores and the cut-points in each screening tool. Due to the sample size, we decided to include the lowest number of variables as possible in order to increase the statistical power (Ranganathan et al., 2017; Norton et al., 2018). Thus, two models were tested for each dependent variable, one including negative body image indicators (i.e., the discrepancy between perceived and ideal body image, negative body attitudes) and another one including positive body image indicators (i.e., body satisfaction, positive body image). Furthermore, we considered as confounding factors only those variables significantly correlated (univariate analyses) with the predicted variables, namely BMI. Given the sample size, bootstrapping (*n* = 1,000) was used for resampling and 95% intervals of confidence were also calculated for each parameter estimation. For assessing goodness of fit of the final models, several tests were considered (i.e., Wald's coefficient, omnibus test of model coefficients and Nagelkerke's test) along with the Hosmer Lemeshow's test, more appropriate for reduced sample size and continuous numerical predictors. Predictive power was considered appropriate when >75% of cases were correctly classified. The SPSS statistical package (IBM SPSS Statistics 25.0, 2017) was used for data analysis. The level of significance was established at *p* < 0.05.

## Results

Table 2 presents the descriptive findings for all the study variables. The participants' average weight was 62.7 ± 13.7 kg, with great between-subjects variability (range = 39–104 kg) due to factors such as age, height and body composition. The average BMI was 23.1 ± 3.9 kg/m^2^, also with great dispersion (range = 16.2–33.6). According to their BMI, 60% of the participants were classified in the normal weight category (19.5–24.9 kg/m^2^), 22.2% in the overweight category (25–29.9 kg/m^2^) and 6.7% in the obesity category (>30 kg/m^2^), while 11.1% were classified as underweight (<19.5 kg/m^2^).

**Table 2:** Descriptive findings and Spearman's *rho* correlations for all the study variables (*N* = 45).

**Variable (possible range of values)**	**M ± DT (range)**	**2**	**3**	**4**	**5**	**6**	**7**	**8**	**9**	**10**	**11**	**12**	**13**	**14**	**15**	**16**	**17**	**18**	**19**	**20**	**21**	**22**
1. BMI	23.1 ± 3.9	−0.61^**^		−0.58^**^	−0.53^**^			0.40^**^	0.58^**^	0.47^**^		0.44^**^	−0.37^*^	0.39^**^				0.30^*^	0.36^*^	0.43^**^	0.50^**^	0.43^**^
	(16.2–33.6)																					
2. Silhouettes PBI	7.7 ± 2.2		0.53^**^	0.69^**^	0.58^**^	−0.37^*^		−0.43^**^	−0.47^**^		−0.36^*^	−0.50^**^	0.40^**^						−0.38^**^		−0.43^**^	−0.30^*^
(1–15)	(4–12)																					
3. Silhouettes IBI	9.8 ± 1.6					−0.27^†^																
(1–15)	(6–12.5)																					
4. PBI-IBI	−2.0 ± 1.8				0.49^**^		−0.26^†^	−0.37^*^	−0.46^**^		−0.33^*^	−0.43^**^	0.33^*^						−0.35^*^	−0.31^*^	−0.44^**^	−0.36^*^
(−14 to 14)	(−6.00 to 1.00)																					
5. BODY	4.9 ± 1.5					−0.45^**^	−0.39^**^	−0.66^**^	−0.73^**^	−0.50^**^	−0.33^*^	−0.74^**^	0.79^**^	−0.49^**^	−0.44^**^		−0.44^**^	−0.34^*^	−0.61^**^	−0.56^**^	−0.72^**^	−0.65^**^
SATISFACTION (1–7)	(2–7)																					
6. BAQ_	2.9 ± 0.7							0.30^*^			0.28^†^	0.43^**^	−0.35^*^								0.27^†^	
Attractiveness (1–5)	(1.6–4.4)																					
7. BAQ_	2.3 ± 0.8							0.58^**^	0.60^**^	0.38^**^		0.72^**^	−0.61^**^	0.66^**^	0.53^**^	0.30^*^	0.60^**^	0.52^**^	0.40^**^	0.67^**^	0.68^**^	0.66^**^
Salience (1–5)	(1.0–4.4)																					
8. BAQ_	1.7 ± 0.6								0.74^**^	0.54^**^	0.28^†^	0.83^**^	−0.74^**^	0.59^**^	0.41^**^	0.34^*^	0.57^**^	0.58^**^	0.49^**^	0.63^**^	0.67^**^	0.69^**^
Disparagement (1–5)	(1.0–3.2)																					
9. BAQ_	2.4 ± 0.9									0.61^**^	0.28^†^	0.91^**^	−0.69^**^	0.61^**^	0.49^**^		0.54^**^	0.46^**^	0.63^**^	0.72^**^	0.78^**^	0.75^**^
Overall obesity (1–5)	(1.2–4.5)																					
10. BAQ_	2.5 ± 0.8											0.63^**^	−0.51^**^	0.50^**^	0.33^*^		0.34^*^	0.47^**^	0.39^**^	0.46^**^	0.52^**^	0.53^**^
Lower obesity (1–5)	(1.3–4.5)																					
11. BAQ_	2.6 ± 0.8											0.46^**^	−0.43^**^									
Fitness (1–5)	(1.2–4.4)																					
12. BAQ_TOTAL	2.4 ± 0.6												−0.80^**^	0.58^**^	0.51^**^	0.27^†^	0.56^**^	0.46^**^	0.54^**^	0.64^**^	0.78^**^	0.71^**^
(1–5)	(1.5–3.7)																					
13. BAS2_TOTAL	3.9 ± 0.9													−0.47^**^	−0.35^*^		−0.37^*^	−0.44^**^	−0.43^**^	−0.54^**^	−0.70^**^	−0.61^**^
(1–5)	(1.7–5.0)																					
14. EAT26_	**0.5 ± 0.5**														0.56^**^	0.48^**^	0.85^**^	0.75^**^	0.49^**^	0.86^**^	0.64^**^	0.80^**^
Restriction (0–3)	(0–2.6)																					
15. EAT26_	0.4 ± 0.6															0.29^†^	0.67^**^	0.36^*^	0.48^**^	0.58^**^	0.60^**^	0.60^**^
Bulimia (0–3)	(0–2.3)																					
16. EAT26_	0.5 ± 0.6																0.78^**^	0.32^*^		0.35^*^	0.27^†^	0.36^*^
Control (0–3)	(0–2.6)																					
17. EAT26_TOTAL	12.1 ± 12.5																	0.59^**^	0.47^**^	0.72^**^	0.62^**^	0.72^**^
(0–78)	(0–64.5)																					
18. CHAD-B_F1	3.0 ± 1.5																		0.52^**^	0.79^**^	0.55^**^	0.81^**^
(1–6)	(1.0–6.0)																					
19. CHAD-B_F2	2.3 ± 1.5																			0.64^**^	0.72^**^	0.82^**^
(1–6)	(1.0–5.6)																					
20. CHAD-B_F3	2.3 ± 1.3																				0.78^**^	0.93^**^
(1–6)	(1.0–6.0)																					
21. CHAD-B_F4	2.6 ± 1.5																					0.89^**^
(1–6)	(1.0–6.0)																					
22. CHAD-B_TOTAL	51.0 ± 25.1																					
(20–120)	(20.0–114.0)																					

** *p < 0.01.*

* *p < 0.05.*

† *p < 0.10*.

The participants identified themselves with an average perceived body image (PBI) corresponding to a small and normal-weight body with little muscle tone, while the average ideal body image (IBI) was notably more athletic (i.e., thin and muscular); therefore, the average PBI-IBI discrepancy corresponded to 2 silhouettes with a higher level of athleticism (Table 2). In terms of percentages, 48.9% of the players chose for PBI silhouettes below the average value, their perceptions corresponding to bodies ranging from overweight/mild obesity (13.3%) to normal weight (35.6%); 26.7% indicated silhouettes corresponding to thin and not muscular bodies; and 24.4% reported to be represented by silhouettes corresponding to slightly to remarkably muscular bodies. Regarding players' ideal body image, 8.9% selected as desirable a figure with overweight and no muscularity, 33.3% wanted their bodies to be slimmer but not toned and 57.8% indicated silhouettes corresponding to larger and more muscular bodies with lower fat mass. The players' body satisfaction was on average moderately high (Table 2), with 62.2% stating that they felt quite or very satisfied with their bodies, 22.2% moderately satisfied and 25.6% not very satisfied. None indicated feeling very little or not satisfied at all with their body and weight.

Regarding their attitudes towards body weight and appearance, the average total score on the BAQ and the subtotals in each dimension were moderate, with no score highlighting as elevated (Table 2). However, comparatively, mean scores were higher for the (Absence of) Attractiveness and (Absence of) Strength/fitness dimensions. Considering their answers in each dimension, ~20% of the participants obtained a score ≥ 3 in any of them, showing negative body attitudes and feelings of self-disparagement and reduced personal worth due to appearance.

Positive body image was also moderately high among the players (Table 2). Whereas, 84.4% of the participants showed a moderate-to-high body appreciation, with scores in the BAS-2 items ≥ 3, a total of 15.6% scored ≤ 3 in any item. Considering their responses to each item, in general the participants reported feeling love and respect for their body and being attentive to their bodies' needs at a higher degree than being able to value their appearance by rejecting the sociocultural standards of beauty, yet the differences in the patterns of scoring were minimal.

Regarding the EAT-26, the participants did not show high scores either in its dimensions (i.e., Restriction, Bulimia and Control) or in the global score (Table 2). Considering the possible range of scores in this instrument, the mean score is apparently not very high, and in fact 84.4% obtained a score < 19 (reduced risk). However, 15.6% reached ≥ 19, the cut-off point for establishing a very high risk. Considering 10 points as an indicative value of existing risk, the players' average score is slightly higher than this value, and in fact 42.2% of the participants scored on this value.

As for the CHAD-B, the subtotal scores are generally low (Table 2), although they are comparatively higher for Fear of gaining weight in periods of rest and use of exercise to lose weight and Excessive concern about body image and dissatisfaction with the body and appearance, compared to Distress in relation to weight and appearance due to the attitudes and comments of other people (e.g., coach, teammates, family, friends) and Obsessive worry about food, diet and weight. The mean global score is below the cut-point for high risk, and 75.6% scored < 66 (reduced risk). However, 31.1% scored ≥ 60 points (notable risk), and 24.4% demonstrated a very high risk for an ED (≥66 points).

In general, the study variables were associated in the expected way (see Table 2). Weight and BMI (*rho* = 0.9, *p* < 0.001) showed the same pattern of correlations with the remaining study variables, and thus only BMI was considered in further analyses. The participants' age was associated only with weight, *rho* = 0.39, *p* < 0.01, and BMI, *rho* = 0.37, *p* < 0.05, so it was not considered for the remaining analyses. Body perceptions towards athleticism (i.e., lower fat and greater muscularity), greater body satisfaction and body appreciation were associated with each other and inversely with BMI, unfavourable self-representations, negative attitudes towards body weight and appearance and risk for eating pathology, which in turn, were directly related to each other. Notably, the correlation between the two instruments used to assess eating symptoms was robust, but in general the CHAD-B showed the above-mentioned associations more clearly than the EAT-26.

Finally, the ORs for eating pathology vulnerability were calculated for both the EAT-26 and the CHAD-B tests. A multivariate logistic regression was conducted for each screening tool including negative body image indicators (i.e., the discrepancy between perceived and ideal body image and total score on negative body attitudes) and positive body image indicators (i.e., body satisfaction and positive body image), controlling for BMI. Table 3 presents the findings. The risk for EDs established with the CHAD-B was predicted by negative attitudes towards the body (BAQ total score), so that higher scores were associated to an odd of ×12-fold of increased risk (OR = 12.3, 95% CI = 5.146–1,831.180), and by body appreciation (BAS-2 total score), so that higher scores were associated with an odd of 83% risk reduction (OR = 0.17, 95% CI = −10.063 to 0.048). Contrarily, only negative body attitudes predicted the risk for EDs as established with the EAT-26, with an odd of ×11-fold of increased risk (OR = 10.9, 95% CI = 0.135–440.794). BMI, the discrepancy between body perceptions and body ideals and body satisfaction were non-significant in establishing the ORs for EDs vulnerability in any model.

**Table 3:** ORs for eating pathology considering both negative and positive body image indicators.

**Criterion**	**Indicators**	**Predictors**	**S.E**.	**Wald's coeff. (*p*)**	**OR**	**95% CI L-U**
EAT-26	Negative BI (1)	BMI	0.140	0.427 (0.508)	0.913	−8.404 to 0.721
		PBI-IBI	0.334	0.976 (0.355)	1.391	–.593 to 114.821
		Negative attitudes	0.987	5.871 **(0.021)**	**10.925**	**0.135 to 440.794**
	Positive BI (2)	BMI	0.124	0.032 (0.879)	0.978	−0.458 to 0.291
		Body satisfaction	0.555	0.027 (0.883)	0.913	−1.599 to 1.583
		Body appreciation	0.825	0.095 (0.745)	0.776	−3.414 to 1.882
CHAD-B	Negative BI (3)	BMI	0.242	1.680 (0.056)	0.731	−122.735 to 16.771
		PBI-IBI	0.437	1.757 (0.067)	1.785	−6.429 to 62.020
		Negative attitudes	2.628	7.333 **(0.002)**	**12.312**	**5.146 to 1,831.180**
	Positive BI (4)	BMI	0.122	0.013 (0.916)	0.986	−0.516 to 0.337
		Body satisfaction	0.582	0.025 (0.868)	0.912	−1.879 to 2.817
		Body appreciation	0.972	3.379 **(0.043)**	**0.168**	–**10.063 to 0.048**

*0 = no risk, 1 = risk. S.E., Standard error; 95% CI, 95% Confidence Interval; L, Lower limit; U, Upper limit. 95% CI not including the value 1 are bolded, noting significant prediction estimations. Bootstrapping n = 1,000. Omnibus test models 1 = χ^2^= 7.887, p < 0.05, 2 = χ^2^= 0.647, p > 0.05, 3 = χ^2^= 30.530, p < 0.001, 4 = χ^2^= 16.464, p = 0.001. Nagelkerke's R^2^ models 1 = 0.28, 2 = 0.03, 3 = 0.73, 4 = 0.46. Hosmer Lemeshow's test models 1 = χ^2^= 10.336, p > 0.05, 2 = χ^2^= 2.125, p > 0.05, 3 = χ^2^= 5.528, p > 0.05, 4 = χ^2^= 5.773, p > 0.05. Predictive power models 1 = 86.7% of cases correctly classified, model 2 = 84.4%, model 3 = 88.9%, model 4 = 82.2%*.

## Discussion

We explored the body-related self-representations and attitudes of amateur female soccer players and their vulnerability to disordered eating behaviours. This study contributes to the research on body image dysfunctions and EDs in Spanish female sports, a context in which around 1 in 10 athletes have symptoms or a high risk for alterations of eating behaviour and body image (Teixidor-Batlle et al., 2021). In addition, it contributes to research on positive body image and its protective role in women, and specifically in sportswomen, an emerging area in which studies are scarce. Moreover, this study contributes to research on women's soccer. Soccer is one of the most popular sports worldwide. Despite the increase in women's participation, soccer continues to be a domain of the male athlete, and female players find many barriers to compete in that territory traditionally constructed as masculine both at a social and a sporting level and to be recognised and valued as athletes (Fisher and Dennehy, 2015; Pfister, 2015; Lunde and Gattario, 2017). However, resisting and defying normative discourses, more and more girls and women are playing soccer at varying levels of performance, from recreational to competitive elite.

We found that the participants' mean BMI corresponded to a normal and “optimal” weight and composition among female soccer players, in line with other findings with Spanish players (Petisco-Rodríguez et al., 2020). Nikolaidis (2014) found in female soccer players in varied high performance levels an average BMI range of 21–24 kg/m^2^ (22.2 ± 0.7), associating a BMI = 22 with an optimal performance (e.g., greater aerobic and anaerobic power, isometric muscle strength, force-speed ratio, flexibility, optimal body composition and general fitness). Likewise, a BMI ≤ 20 and ≥25 entails reduced performance, although it may also have certain advantages (e.g., in terms of speed or strength-power, respectively). The importance of considering body composition instead of BMI or weight has been stressed repeatedly in terms of athletic performance achievement and athletes' optimal health (Kasper et al., 2021). Nevertheless, BMI was used in the present study because a body composition exploration was not possible, and self-reported weight and height were instead easily collected. In addition, BMI can be very useful in athletes with underweight and serves to warn them regarding conditions due to low weight (e.g., disordered eating, low energy availability, female athlete triad/relative energy deficiency syndrome) that can impair performance and well-being.

As for perceived and ideal body image and body satisfaction, the participants as a group reported body perceptions that coincide with their sporting bodies, although they would like to have more athletic bodies, i.e., with lower fat and higher muscularity, corporealities with a higher conformity to the physical demands of their sport. With the same silhouette-based tool, it has been found that Spanish young women (18–40 years) from the general population have an average body image of 6.8 ± 2.4, corresponding to self-perceptions of slightly overweight bodies with little muscle tone or definition, and an ideal body image corresponding to a figure two silhouettes thinner and more toned (Ramírez et al., 2015). In brief, athletes show self-representations of their bodies that correspond to their reality as athletes compared to non-athletes counterparts, but their body demands are also greater in terms of muscularity and low percentage of fat mass, in association with the functionality of their body and athletic activity (Fisher and Dennehy, 2015; Heinecken, 2015; Devonport et al., 2019). However, it is important to recognise the diversity of self-perceptions and body ideals, namely not all of them had body perceptions of an athletic body, nor all of them wished a more athletic body.

The participants reported having a fairly high level of body satisfaction. Compared to the body satisfaction of Spanish non-athlete young women (4.0 ± 1.5 in the same 1-to-7-point scale) (Ramírez et al., 2015), female athletes are more satisfied with their bodies. Different studies and meta-analysis support that female athletes have better body perceptions and greater body satisfaction compared to non-athlete women (Sabiston et al., 2019), suggesting that women who participate in sports may encounter unique benefits in terms of body image, as they experience the embodiment of the activity and the physical changes related to performance, allowing them to appreciate body functionality over appearance; moreover, a shift in beauty ideals may occur as a larger body size is approved, the acceptance and celebration of bodies diversity is promoted and self-objectification is reduced (Heinecken, 2015; Liechty et al., 2015; Lunde and Gattario, 2017). However, even when having positive self-perceptions, they are not protected for having dysfunctional attitudes towards the body, weight and appearance and unhealthy management practises. Female athletes face particular internal and external performance pressures, body- and weight-related pressures, increased public exposure of the body, common regulatory external practises such as a strict control of food and weight –sometimes in public weigh-ins–, rigid beliefs on the relationship between weight and maximum performance, devaluation by the media, etc. Additionally, athletes have to continually, on and off the field, negotiate their femininity as it is embodied in their athletic bodies (Fisher and Dennehy, 2015; Liechty et al., 2015; Bennett et al., 2016; Lunde and Gattario, 2017; Allan and Owen, 2019; Cosh et al., 2019; Devonport et al., 2019), and they are subject of greater social (sexual) objectification, which increases a focus on appearance, thin-ideal internalisation and body shame and surveillance (Varnes et al., 2015).

Therefore, the results obtained in terms of their body attitudes are not surprising. Although their average scores on the BAQ differ considerably from the scores observed in young women with anorexia or bulimia and are similar to those of healthy females (Probst et al., 2008; de Souza et al., 2014), 20% of the participants showed negative attitudes towards their weight and body and feelings of contempt and lack of personal worth due to appearance, which is consistent with the prevalence observed in the present study for body dissatisfaction as well as with the percentage of players with the worst body perceptions and the greatest discrepancy between their perceived and ideal bodies. Women with disordered eating show significantly higher scores on the BAQ and its dimensions and this self-report—particularly the subdimensions of attractiveness, strength/fitness and self-disparagement—allows discriminating between women with EDs and healthy females (Probst et al., 2008). To our knowledge, this questionnaire has not previously been used with athletes, so this study can make a contribution in this regard. Nevertheless, it has not been used either with Spanish females, and thus more research is needed to support its usefulness in Spanish and athletic samples.

Body attitudes can be harmful when they are centred on weight and appearance and are a result of the internalisation of the sociocultural beauty canons and the subsequent body objectification, producing feelings of shame, fear, concealment, insecurity, worry and distress pivoting on food and body concerns and unhealthy control strategies. On the contrary, healthy and positive attitudes towards the body are based on body respect, acceptance, appreciation and care from a critical personal position confronting social pressures (Tylka, 2018). They are associated with feelings of satisfaction, recognition, pride, love, de-objectification and overall well-being and are translated into a relationship with food and with the body with attention to its needs, and thus a higher use of healthy self-caring strategies; moreover, being self-compassionate and confident with their bodies, respecting and treating their bodies with kindness, the athletes can experience higher body satisfaction and a shift to an adaptive focus on functionality (Killham et al., 2015; Lunde and Gattario, 2017; Eke et al., 2020). In our study, the participants reported a moderately high body appreciation on average, particularly in terms of feeling love and respect for their body and being attentive to their bodies' needs. Their scores were similar to those of other female athletes (Soulliard et al., 2019; Voelker et al., 2019). Their scores were also similar to those of young and adult Spanish non-athlete women (Swami et al., 2017), which is contrary to expectations, since recent research suggests that focusing on body functionality instead of appearance-related features can improve body appreciation (Alleva and Tylka, 2021). Noteworthy, 1 in 6 players had low positive body image. It has been argued that participation in sport can be a rewarding experience for women, protecting them against negative self-perceptions and body attitudes and problematic eating behaviours, but it can also involve unpleasant experiences that translate into threats to body image by reinforcing negative self-thoughts and behaviours, ultimately affecting athletes' well-being (Fisher and Dennehy, 2015; Lunde and Gattario, 2017; Cosh et al., 2019; Devonport et al., 2019; Eke et al., 2020). Research highlights the need to better understand the experiences of women in the sports environment to obtain valuable information on how to make the sport context a positive scenario for females' body image (Lunde and Gattario, 2017).

On the other hand, the risk for eating pathology is elevated among young females (Lindvall Dahlgren et al., 2017; Qian et al., 2021). The social pressures on women's bodies are increasing, and currently girls and women must conform the slim and toned expectations known as the ideal athletic body, to be socially accepted (Hartmann et al., 2018; Henn et al., 2019; Aanesen et al., 2020; Lang and Rancourt, 2020; Steinfeld et al., 2020). These pressures are rooted in the prevailing sociocultural, patriarchal canons of feminine beauty, transmitted through all channels and considered valid, accepted and internalised, guiding females' experiences. The athletic ideal has thus disciplinary effects (Aanesen et al., 2020). The athletic ideal is linked to higher body dissatisfaction than the thin ideal, as it is more exigent and less attainable (Betz and Ramsey, 2017). In athletes, body-related pressures are higher than in the general population, since sportswomen have also to face the tensions arising from the pressures for results, the belief that low weight leads to better performance, the reality that weight and appearance are sometimes relevant for athletic performance (e.g., weight-classes and aesthetic sports), the constant visibility of the body (e.g., uniforms) and the continuous scrutiny of the body (e.g., coaches, public weigh-ins, weight control strategies, fans or the sports press). Therefore, the risk for disordered eating and EDs is considerably higher among athletes compared to non-athletes (Dilyara and Zuzanna, 2016; Joy et al., 2016; Díaz et al., 2018; Mancine et al., 2020), particularly in sports emphasising weight and appearance. One of the most extensive and cited reviews (Bratland-Sanda and Sundgot-Borgen, 2013) indicates prevalence rates of subclinical eating pathologies and manifestations of altered behaviours among female athletes of 20–62%, and of clinical EDs of 6–45%. As repeatedly pointed out, these figures probably underestimate the real numbers, since dysfunctional behaviours and symptoms tend to be hidden or denied for fear of reprisals or stigma (Joy et al., 2016). Specifically on ball sports such as soccer, basketball, handball, etc., there is currently growing research on body perceptions, body satisfaction and risk for EDs. Body and eating issues in these sports are more common and frequent than expected (Díaz et al., 2018), such that the label of “low risk” sports does not fit with the reality of athletes.

In the present study, about 2 in 10 athletes are at risk for, or are already suffering from, eating pathology. It is a very high and, unfortunately, very common number in sports, also among Spanish female athletes, although other studies report lower prevalence rates (Godoy-Izquierdo et al., 2019a; Petisco-Rodríguez et al., 2020; Teixidor-Batlle et al., 2021). Compared to males, there is double or even triple incidence and prevalence rates among females (Teixidor-Batlle et al., 2021). Recent research on EDs in Spanish athletes of both sexes-genders and different levels of performance and sports modalities concludes that ~5–10% have a high vulnerability to an ED (Teixidor-Batlle et al., 2021), including soccer players (Godoy-Izquierdo et al., 2019a). These findings coincide with other previous studies carried out with elite athletes from national high-performance centres (Dosil et al., 2012). Specifically in women, 8% of the athletes showed high risk in the studies by Dosil et al. and Teixidor-Batlle et al., with a prevalence among them 3 times higher than that of male athletes in both studies. In the most recent of both studies, female athletes in ball sports showed a prevalence of 4%, compared to 13–14% in aesthetic and technical sports, yet prevalence was not explored specifically for soccer. In the study by Godoy-Izquierdo et al., the authors performed a cluster analysis to detect risk configurations and identified three profiles of high risk (8.7%), moderate risk (45.1%) and low risk (46.2%), and found that the soccer players showed a profile very similar to that of the moderate, but existent, risk cluster. Furthermore, almost 2/3 of the soccer players were grouped into moderate and high risk clusters. Although this study did not explore differences by sex-gender (36% of the total sample and 15% of the subsample of soccer players were women), the authors found, considering all participating athletes, that low-risk and moderate-risk clusters were made up of twice as many males as females. More recently, Petisco-Rodríguez et al. (2020) have found that between 5 and 12.5% (depending on the screening instrument) of adolescent and young female soccer players have a high risk for EDs, these figures being considerably higher than those observed in gymnasts, but lower than those of non-athlete controls. Studies carried out with non-Spanish female soccer players indicate a prevalence of 11%, also lower than those found among non-athlete girls and women (Abbott et al., 2021); others indicate that while only 0.5% are at high risk, an additional 7.7% could be at moderate risk (Prather et al., 2016). Studies such as these support questioning classifications of athletes into risk gradients attending exclusively to the sport modality (i.e., high and low risk sports) (Díaz et al., 2018) and highlight the need to consider mainly individual factors (e.g., beliefs and attitudes regarding weight and appearance, unhealthy weight management behaviours) and contextual factors (e.g., pressures from coaches and teammates, sociocultural pressures on the aesthetic body and the athletic femenine vs. masculinised body), as well as intersectional factors, gender in particular (Chang et al., 2020).

It is interesting to note how the two screening self-reports, one of them general and the other specific for sport, detected potential clinical cases. According to the CHAD-B, between 11 and 14 players were at high or moderately high risk for eating pathology, respectively; according to the EAT-26, 7 players were at risk. Both instruments coincide in 6 participants, 5 athletes are detected by CHAD-B but not by EAT-26, and 1 was detected by EAT-26 but not by CHAD-B. It seems clear then that assessment instruments specifically designed for the sports context, which take into account particular risk factors and eating pathology manifestations, are more sensitive to detect vulnerability than tools designed for the general population (with or without eating symptoms) (Pope et al., 2015).

More importantly, we found that the vulnerability for eating pathology was determined mainly by both negative and positive attitudes towards the body, irrespective of body perceptions and body satisfaction as well as BMI. Among the two screening tools, the CHAD-B demonstrated the most interesting findings. After controlling for confounders, those players holding more negative attitudes towards their body were more likely to report eating subclinical or clinical pathology. The odds for them developing an ED was about 12 times as high compared with athletes with lower negative attitudes regarding their body weight and shape (OR = 12.3, *p* < 0.01). On the contrary, those athletes celebrating their bodies, caring for them and resisting the sociocultural pressures of beauty were less likely to suffer from eating and body image pathologies. The odds for them developing an ED was >80% lower compared with athletes with lower positive attitudes regarding their body (OR = 0.17, *p* < 0.05). Nevertheless, there is still a noticeable part of the variance which remained unexplained, pointing to the need of considering other factors by future research in order to fully understand the risk for eating pathology in female sports. The results of this study support the protective role of positive body image in preventing dysfunctional eating and contribute novel findings in sports context, with potential implications for coaches, nutritionists and psychologists to encourage a culture that focuses less on body weight and appearance and more on cultivating body positivity.

Our findings have interesting practical applications. Given the high incidence and prevalence of EDs in the sports context, it is necessary that all instances related to sports activity take responsibility and that multidisciplinary preventive and therapeutic programs including psychological and nutritional counselling are carried out in order to reduce current rates (Bar et al., 2016; Joy et al., 2016; de Bruin, 2017; Voelker and Reel, 2018; Voelker and Galli, 2019; Chang et al., 2020; Sandgren et al., 2020). Previous evidence indicates that despite increasing awareness on the importance of an adequate diet for better athletic performance, gender stereotypes continue to bother female athletes, influencing their nutritional choices and limiting their food intake (for instance, yet they are attentive to their increased needs, they report feeling shame for eating “too much” when compared to non-athlete females; Lunde and Gattario, 2017). This is further linked to social and athletic prejudices against overweight as well as the athletes' struggle for attaining a functional body or a physically attractive body according to the hegemonic beauty ideals, with many athletes reporting feeling that they are unable to adjust to both ideals at the same time (Lunde and Gattario, 2017). Furthermore, the sports subculture, namely the “normative” beliefs and practises accepted and expected within the soccer subculture, as well as dietary practises and preferences in everyday life (e.g., sociocultural and family values) collide with nutritional recommendations for athletes, and this causes them to find a conflict regarding their diet as an athlete or as a woman (Ono et al., 2012). In addition, the influences of coaches and teammates on athletes' health choices, including behaviours for weight and appearance regulation, have also been stressed (Beckner and Record, 2016; Bennett et al., 2016; Díaz et al., 2019).

An alternative to traditional proposals derived from dominant gendered cultures and subcultures of appearance and food should be a priority in sports. With a focus on the pursuit of improving athletes' health and performance, some recent attempts (e.g., Tylka and Homan, 2015; Voelker et al., 2019, 2021) have been conducted based on the non-weight-and-appearance-centred paradigm of positive body image and intuitive eating. By deconstructing concepts of beauty, weight, food and performance, unmasking myths and highlighting the importance of approaching psychological, emotional and physical health in a more holistic, friendly, flexible and integrative way, based on a person-as-a-whole principle, this new approach can help athletes to listen to their bodies and to identify their internal signals to guide their food intake, thus achieving better performance. Given that positive body image interventions are helpful for women (Guest et al., 2019), instructing athletes on positive ways of inhabiting the body can be promising in EDs prevention in the sports contexts (Piran, 2015; Wood-Barcalow and Augustus-Horvath, 2018). Given that a healthy diet adapted to the condition of each athlete is a requirement for maximum performance (FIFA, 2010; Escalante, 2016; Thomas et al., 2016; Gastrich et al., 2020), providing athletes an adequate nutrition education in sports combined with an individualised training in intuitive eating (Oh et al., 2012; Plateau et al., 2017) and positive body image in the sports context could be a promising approach for increasing athletes' well-being and performance while also decreasing their risk for body image and eating pathology.

Despite the contributions of this study, it is important to stress its limitations, so that future research can adequately address them. First, the sample is small, given that it was composed of those players who willingly accepted to participate among all the invited players; although this was our interest, it is also limited to female soccer players. Thus, the present study has to be considered as a preliminary report with limited generalizability of the findings, and more research is needed with women from this and other sports, of different performance levels and from other nations. Second, only self-reports have been used to assess the study variables including the risk for eating pathology, instead of other more appropriate strategies for the diagnosis of EDs (i.e., clinical interview). In addition, athletes may have underreported their unhealthy attitudes and practises due to the non-anonymous nature of the evaluation, or contrarily these might have been overreported due to the possibility that the participants were those players with higher concerns on these issues. Third, some of the assessment tools have been scarcely used to date in the Spanish population or athletic females. More research is needed to confirm their utility with these samples. In addition, yet previously supported by other researchers (Pope et al., 2015; Rodríguez et al., 2015), it is necessary to confirm that standardised screening tools such as the EAT-26 must be substituted with specific tools such as the CHAD-B for a more suited identification of eating pathology vulnerability in the sports context. Moreover, other important dimensions (e.g., altered but normal behaviours in sport) or influencing factors (e.g., pressure from the coach or teammates) have not been considered. These factors may explain the *what, when and how* certain attitudes and practises regarding weight and body management become problematic in the sporting context, and should be considered by future research. Previous research indicates that variables such as being an athlete, sex-gender, age, educational level, sport modality or level of performance can be important factors regarding nutritional knowledge and food intake, since they can be associated with aspects such as a greater interest in performance or greater availability of resources, although the evidence is not conclusive for any of these factors (Trakman et al., 2016). Due to the numbers in the present study, we were not able to analyse the possible influence of these factors. Finally, the correlational and cross-sectional nature of this study prevents us from drawing conclusions about possible cause-effect relationships; more suitable designs (e.g., longitudinal, experimental) and other statistical analyses can offer more conclusive answers to our research questions. Moreover, the nature of the study impeded us to explore more deeply the players' conflict with their athletic and aesthetic bodies, the performativity of athleticism and femininity and the balances with instrumental-functional and appearance-social dimensions of their body image. Other methodologies, such as qualitative designs, are more appropriate for understanding the complexities of body image in female soccer players.

In conclusion, the soccer players in general showed self-representations of their bodies that corresponded to their reality as athletes, but their body ideals were also more demanding in terms of low fat and muscularity, in association with the functionality of their body and their athletic activity. Despite having a fairly high body satisfaction, they expressed a desire to adjust more to the ideal body determined by the physical demands of soccer. Interindividual variability was also observed and is worthy to be noted. Although sports such as soccer have been traditionally classified as low risk for having a disorder related to body image and eating behaviour, we found that around 2 in 10 players were at risk of suffering from an ED, which is related mainly to their negative and unhealthy body attitudes. Players with negative attitudes towards their bodies were an odd 12 times more likely to develop an ED compared to those with lower self-devaluation, after adjusting for BMI and body perceptions. On the contrary, players who appreciate their bodies and hold a positive body image had an odd 0.17 times likely to suffer from eating pathology, after adjusting for BMI and body satisfaction. Our results point to the need to change the conceptual paradigm that guides the interventions for the prevention and management of dysfunctions related to body image and eating behaviour in sportswomen, among which the positive body image paradigm appears as a promising approach.

## Data Availability Statement

The raw data supporting the conclusions of this article will be made available by the authors, without undue reservation.

## Ethics Statement

The studies involving human participants were reviewed and approved by Ethic Commitee of University of Granada. Written informed consent to participate in this study was provided by the participants, or the participants' legal guardian/next of kin.

## Author Contributions

DG-I and ID: conceptualisation, data curation, investigation, methodology, project administration, resources, supervision, and writing—review and editing. DG-I: formal analysis, funding acquisition, and writing—original draught. Both authors have read and agreed to the published version of the manuscript.

## Funding

This research was financed by the economic support by the Junta de Andalucía (Spain) to the Research Group CTS267 Health Psychology/Behavioural Medicine.

## Conflict of Interest

The authors declare that the research was conducted in the absence of any commercial or financial relationships that could be construed as a potential conflict of interest.

## Publisher's Note

All claims expressed in this article are solely those of the authors and do not necessarily represent those of their affiliated organizations, or those of the publisher, the editors and the reviewers. Any product that may be evaluated in this article, or claim that may be made by its manufacturer, is not guaranteed or endorsed by the publisher.

## References

[B1] Aanesen S. M. Notøy R. R. G. Berg H. (2020). The re-shaping of bodies: a discourse analysis of feminine athleticism. Front. Psychol. 11:1751. 10.3389/fpsyg.2020.01751 32793072 PMC7394218

[B2] Abbott W. Brett A. Brownlee T. E. Hammond K. M. Harper L. D. Naughton R. J.. (2021). The prevalence of disordered eating in elite male and female soccer players. Eating Weight Disord. Stud. Anorexia Bulimia Obes. 26, 491–498. 10.1007/s40519-020-00872-0 32107745 PMC7946675

[B3] Allan T. Owen A. L. (2019). “For athletes, there are many pressures to be strong and fit, but also have that feminine look”: a study of female athletes' body image. J. Qual. Res. Sport Stud. 13, 85–96.

[B4] Alleva J. M. Tylka T. L. (2021). Body functionality: a review of the literature. Body Image 36, 149–171. 10.1016/j.bodyim.2020.11.006 33321273

[B5] Andrew R. Tiggemann M. Clark L. (2016). Predicting body appreciation in young women: an integrated model of positive body image. Body Image 18, 34–42. 10.1016/j.bodyim.2016.04.003 27240100

[B6] Bar R. J. Cassin S. E. Dionne M. M. (2016). Eating disorder prevention initiatives for athletes: a review. Eur. J. Sport Sci. 16, 325–335. 10.1080/17461391.2015.1013995 25815432

[B7] Beckner B. N. Record R. A. (2016). Navigating the thin-ideal in an athletic world: influence of coach communication on female athletes' body image and health choices. Health Commun. 31, 364–373. 10.1080/10410236.2014.957998 26361233

[B8] Bennett E. V. Scarlett L. Hurd Clarke L. Crocker P. R. E. (2016). Negotiating (athletic) femininity: the body and identity in elite female basketball players. Qual. Res. Sport Exercise Health 9, 233–246. 10.1080/2159676X.2016.1246470

[B9] Ben-Tovim D. I. Walker M. K. (1991). The development of the Ben-Tovim Walker Body Attitudes Questionnaire (BAQ), a new measure of women's attitudes towards their own bodies. Psychol. Med. 21, 775–784. 10.1017/S0033291700022406 1946865

[B10] Betz D. E. Ramsey L. R. (2017). Should women be “all about that bass”?: Diverse body-ideal messages and women's body image. Body Image 22, 18–31. 10.1016/j.bodyim.2017.04.004 28554090

[B11] Bratland-Sanda S. Sundgot-Borgen J. (2013). Eating disorders in athletes: overview of prevalence, risk factors and recommendations for prevention and treatment. Eur. J. Sport Sci. 13, 499–508. 10.1080/17461391.2012.740504 24050467

[B12] Busanich R. McGannon K. R. (2010). Deconstructing disordered eating: a feminist psychological approach to the body, food, and exercise relationship in female athletes. Quest 62, 385–405. 10.1080/00336297.2010.10483656

[B13] Butler J. (1990). Gender Trouble: Feminism and the Subversion of Identity. Abingdon: Routledge.

[B14] Cash T. F. Pruzinsky T. (2002). Body Image: A Handbook of Theory, Research, and Clinical Practice. New York, NY: Guilford Press.

[B15] Chang C. Putukian M. Aerni G. Diamond A. Hong G. Ingram Y.. (2020). Mental health issues and psychological factors in athletes: detection, management, effect on performance and prevention: American Medical Society for Sports Medicine Position Statement—executive summary. Br. J. Sports Med. 54, 216–220. 10.1136/bjsports-2019-101583 31810972

[B16] Cosh S. Tully P. J. Crabb S. (2019). Discursive practices around the body of the female athlete: an analysis of sport psychology interactions in elite sport. Psychol. Sport Exerc. 43, 90–104. 10.1016/j.psychsport.2018.12.021

[B17] de Bruin A. P. Oudejans R. R. (2018). Athletes' body talk: the role of contextual body image in eating disorders as seen through the eyes of elite women athletes. J. Clin. Sport Psychol. 12, 675–698. 10.1123/jcsp.2018-0047

[B18] de Bruin A. P. Oudejans R. R. D. Bakker F. C. Woertman L. (2011). Contextual body image and athletes' disordered eating: the contribution of athletic body image to disordered eating in high performance women athletes. Eur. Eating Disord. Rev. 19, 201–215. 10.1002/erv.1112 21584913

[B19] de Bruin A. P. K. (2017). Athletes with eating disorder symptomatology, a specific population with specific needs. Curr. Opin. Psychol. 16, 148–153. 10.1016/j.copsyc.2017.05.009 28813340

[B20] de Souza A. C. Pisciolaro F. Polacow V. O. Cordás T. A. Alvarenga M. dos S. (2014). Atitudes em relação ao corpo e à alimentação de pacientes com anorexia e bulimia nervosa. J. Bras. Psiquiatr. 63, 1–7. 10.1590/0047-2085000000001

[B21] Devonport T. J. Russell K. Leflay K. Conway J. (2019). Gendered performances and identity construction among UK female soccer players and netballers: a comparative study. Sport Soc. 22, 1131–1147. 10.1080/17430437.2018.1504773

[B22] Díaz I. Godoy-Izquierdo D. Navarrón E. Ramírez M. J. Dosil J. (2018). Eating disorders in sports and football: an updated review. Cuadernos de Psicología del Deporte 18, 45–56.

[B23] Díaz I. Ramírez M. J. Navarrón E. Godoy-Izquierdo D. (2019). Creencias, actitudes y conductas de riesgo de entrenadores en relación con el peso de sus deportistas: un estudio descriptivo. Revista de Psicología Aplicada al Deporte y al Ejercicio Físico 4, 1–8. 10.5093/rpadef2019a10

[B24] Dilyara I. Zuzanna G. (2016). Eating disorders in sport: Review of prevalence, risk factors, and studies of eating disorders in highly competing athletes. J. Educ. Health Sport 6, 351–358. 10.5281/zenodo.55703

[B25] Dosil J. Díaz I. Viñolas A. Díaz O. (2012). Prevención y detección de los trastornos de alimentación en deportistas de alto rendimiento (CAR, CEARE y CTD). Cuadernos de Psicología del Deporte 12, 163–166. 10.4321/S1578-84232012000100019

[B26] Eke A. Adam M. Kowalski K. Ferguson L. (2020). Narratives of adolescent women athletes' body self-compassion, performance and emotional well-being. Qual. Res. Sport Exercise Health 12, 175–191. 10.1080/2159676X.2019.1628805

[B27] Escalante G. (2016). Nutritional considerations for female athletes. Strength Cond. J. 38, 57–63. 10.1519/SSC.0000000000000203

[B28] FIFA (2010). Nutrition for Footbal. A Practical Guide to Eating and Drinking for Health and Performance. Zurich, Switzerland: F-MARC, FIFA Production.

[B29] Fisher C. D. Dennehy J. (2015). Body projects: making, remaking, and inhabiting the woman's futebol body in Brazil. Sport Soc. 18, 995–1008. 10.1080/17430437.2014.997578

[B30] Folscher L. L. Grant C. C. Fletcher L. van Rensberg D. C. (2015). Ultra-marathon athletes at risk for the female athlete triad. Sports Med. Open 1, 1–8. 10.1186/s40798-015-0027-7 26380807 PMC4564455

[B31] Gardner R. M. Brown D. L. (2010). Body image assessment: a review of figural drawing scales. Pers. Individ. Dif. 48, 107–111. 10.1016/j.paid.2009.08.017

[B32] Garner D. M. Olmsted M. P. Bohr Y. Garfinkel P. E. (1982). The eating attitudes test: psychometric features and clinical correlates. Psychol. Med. 12, 871–878. 10.1017/S0033291700049163 6961471

[B33] Gastrich M. D. Quick V. Bachmann G. Moriarty A. M. D. (2020). Nutritional risks among female athletes. J. Womens Health 29, 693–702. 10.1089/jwh.2019.8180 32040354

[B34] Godoy-Izquierdo D. Díaz I. Ramírez M. J. Navarrón E. Dosil J. (2019a). Risk for eating disorders in “high”- and “low”-risk sports and football (soccer): a profile analysis with clustering techniques. Revista de Psicología del Deporte 28, 117–126.

[B35] Godoy-Izquierdo D. Navarrón E. Ramírez M. Díaz I. Ogallar A. Lara R. (2019b). Impacto de las actitudes sexistas en las percepciones corporales y el riesgo de trastornos de la conducta alimentaria en mujeres y hombres deportistas, in XVI Congreso Nacional de Psicología de la Actividad Física y el Deporte. Valencia, España.

[B36] Godoy-Izquierdo D. Ramírez M. Navarrón E. Díaz I. (2017). Predictores del riesgo de trastornos relacionados con la alimentación y la conducta física en deportistas de diversas modalidades deportivas, in XV Congreso Andaluz y II Luso-Andaluz de Psicología de la Actividad Física y el Deporte. Granada, España.

[B37] Grogan S. (2017). Body Image: Understanding Body Dissatisfaction in Men, Women, and Children. 3rd ed. New York, NY: Routledge.

[B38] Guest E. Costa B. Williamson H. Meyrick J. Halliwell E. Harcourt D. (2019). The effectiveness of interventions aiming to promote positive body image in adults: a systematic review. Body Image 30, 10–25. 10.1016/j.bodyim.2019.04.002 31077956

[B39] Hartmann A. S. Steenbergen F. Vocks S. Büsch D. Waldorf M. (2018). How healthy is a desire to be fit and strong? Drives for thinness, leanness, and muscularity in women in weight training. J. Clin. Sport Psychol. 12, 544–561. 10.1123/jcsp.2018-0022

[B40] Heinecken D. (2015). “So tight in the thighs, so loose in the waist”: embodying the female athlete online. Femin. Media Stud. 15, 1035–1052. 10.1080/14680777.2015.1033638

[B41] Henn A. T. Taube C. O. Vocks S. Hartmann A. S. (2019). Body image as well as eating disorder and body dysmorphic disorder symptoms in heterosexual, homosexual, and bisexual women. Front. Psychiatry 10:531. 10.3389/fpsyt.2019.00531 31427996 PMC6689822

[B42] Heradstveit O. Hysing M. Nilsen S. A. Bøe T. (2020). Symptoms of disordered eating and participation in individual-and team sports: a population-based study of adolescents. Eat. Behav. 39:101434. 10.1016/j.eatbeh.2020.101434 32980592

[B43] Homan K. J. Tylka T. L. (2015). Self-compassion moderates body comparison and appearance self-worth's inverse relationships with body appreciation. Body Image 15, 1–7. 10.1016/j.bodyim.2015.04.007 25978272

[B44] Jankauskiene R. Baceviciene M. Trinkuniene L. (2020). Examining body appreciation and disordered eating in adolescents of different sports practice: cross-sectional study. Int. J. Environ. Res. Public Health 17:4044. 10.3390/ijerph17114044 32517115 PMC7312658

[B45] Joy E. Kussman A. Nattiv A. (2016). 2016 update on eating disorders in athletes: a comprehensive narrative review with a focus on clinical assessment and management. Br. J. Sports Med. 50, 154–162. 10.1136/bjsports-2015-095735 26782763

[B46] Kampouri D. Kotopoulea-Nikolaidi M. Daskou S. Giannopoulou I. (2019). Prevalence of disordered eating in elite female athletes in team sports in Greece. Eur. J. Sport Sci. 19, 1267–1275. 10.1080/17461391.2019.1587520 30880593

[B47] Kantanista A. Glapa A. Banio A. Firek W. Ingarden A. Malchrowicz-Mośko E.. (2018). Body image of highly trained female athletes engaged in different types of sport. Biomed Res. Int. 2018:6835751. 10.1155/2018/6835751 29662894 PMC5831824

[B48] Kasper A. M. Langan-Evans C. Hudson J. F. Brownlee T. E. Harper L. D. Naughton R. J.. (2021). Come back skinfolds, all is forgiven: a narrative review of the efficacy of common body composition methods in applied sports practice. Nutrients 13:1075. 10.3390/nu13041075 33806245 PMC8065383

[B49] Killham M. E. Kowalski K. C. Duckham R. L. (2015). Self-compassion and women athletes' body appreciation and intuitive eating experiences. J. Exercise Mov. Sport 47:90.

[B50] Kong P. Harris L. M. (2014). The sporting body: body image and eating disorder symptomatology among female athletes from leanness focused and nonleanness focused sports. J. Psychol. Interdiscip. Appl. 149, 141–160. 10.1080/00223980.2013.846291 25511202

[B51] Krane V. Choi P. Y. Baird S. M. Aimar C. M. Kauer K. J. (2004). Living the paradox: female athletes negotiate femininity and muscularity. Sex Roles 50, 315–329. 10.1023/B:SERS.0000018888.48437.4f

[B52] Krane V. Waldron J. Michalenok J. Stiles-Shipley J. (2001). Body image concerns in female exercisers and athletes: a feminist cultural studies perspective. Women Sport Phys. Act. J. 10, 17–54. 10.1123/wspaj.10.1.17

[B53] Lang B. Rancourt D. (2020). Drive for leanness: potentially less maladaptive compared to drives for thinness and muscularity. Eating Weight Disord. Stud. Anorexia Bulimia Obesity 25, 1213–1223. 10.1007/s40519-019-00753-1 31342456

[B54] Liechty T. Sveinson K. Willfong F. Evans K. (2015). “It doesn't matter how big or small you are… there's a position for you”: body image among female tackle football players. Leisure Sci. 37, 109–124. 10.1080/01490400.2014.980591

[B55] Lindvall Dahlgren C. Wisting L. R.ø Ø. (2017). Feeding and eating disorders in the DSM-5 era: a systematic review of prevalence rates in non-clinical male and female samples. J. Eating Disord. 5:56. 10.1186/s40337-017-0186-7 29299311 PMC5745658

[B56] Lunde C. Gattario K. H. (2017). Performance or appearance? Young female sport participants' body negotiations. Body Image 21, 81–89. 10.1016/j.bodyim.2017.03.001 28365534

[B57] Mancine R. P. Gusfa D. W. Moshrefi A. Kennedy S. F. (2020). Prevalence of disordered eating in athletes categorized by emphasis on leanness and activity type - a systematic review. J. Eating Disord. 8:47. 10.1186/s40337-020-00323-2 33005418 PMC7523350

[B58] Mathisen T. F. Sundgot-Borgen J. (2019). Mental health symptoms related to body shape idealization in female fitness physique athletes. Sports 7:236. 10.3390/sports7110236 31739479 PMC6915661

[B59] Menzel J. E. Levine M. P. (2011). Embodying experiences and the promotion of positive body image: the example of competitive athletics, in Self-Objectification in Women: Causes, Consequences, and Counteractions, eds Calogero R. M. Tantleff-Dunn S. Thompson J. K. (Washington, DC: American Psychological Association), 163–186.

[B60] Neves C. M. Filgueiras Meireles J. F. Berbert de Carvalho P. H. Schubring A. Barker-Ruchti N. Caputo Ferreira M. E. (2017). Body dissatisfaction in women's artistic gymnastics: a longitudinal study of psychosocial indicators. J. Sports Sci. 35, 1745–1751. 10.1080/02640414.2016.1235794 27690759

[B61] Nikolaidis P. T. (2014). Weight status and physical fitness in female soccer players: is there an optimal BMI? Sport Sci. Health 10, 41–48. 10.1007/s11332-014-0172-2

[B62] Norton E. C. Dowd B. E. Maciejewski M. L. (2018). Odds ratios–current best practice and use. JAMA 320, 84–85. 10.1001/jama.2018.6971 29971384

[B63] Oh K. H. Wiseman M. C. Hendrickson J. Phillips J. C. Hayden E. W. (2012). Testing the acceptance model of intuitive eating with college women athletes. Psychol. Women Q. 36, 88–98. 10.1177/0361684311433282

[B64] Ono M. Kennedy E. Reeves S. Cronin L. (2012). Nutrition and culture in professional football. A mixed method approach. Appetite 58, 98–104. 10.1016/j.appet.2011.10.007 22027271

[B65] Perry C. Champ F. M. Macbeth J. Spandler H. (2021). Mental health and elite female athletes: a scoping review. Psychol. Sport Exerc. 56:101961. 10.1016/j.psychsport.2021.101961

[B66] Petisco-Rodríguez C. Sánchez-Sánchez L. C. Fernández-García R. Sánchez-Sánchez J. García-Montes J. M. (2020). Disordered eating attitudes, anxiety, self-esteem and perfectionism in young athletes and non-athletes. Int. J. Environ. Res. Public Health 17:6754. 10.3390/ijerph17186754 32948005 PMC7559299

[B67] Petrie T. A. Greenleaf C. A. (2012). Eating disorders in sport, in The Oxford Handbook of Sport and Performance Psychology, ed Murphy S. (New York, NY: Oxford University Press), 635–659.

[B68] Pfister G. (2015). Assessing the sociology of sport: on women and football. Int. Rev. Sociol. Sport 50, 563–569. 10.1177/1012690214566646

[B69] Piran N. (2015). New possibilities in the prevention of eating disorders: the introduction of positive body image measures. Body Image 14, 146–157. 10.1016/j.bodyim.2015.03.008 25886711

[B70] Plateau C. R. Petrie T. A. Papathomas A. (2017). Learning to eat again: intuitive eating practices among retired female collegiate athletes. Eat. Disord. 25, 92–98. 10.1080/10640266.2016.1219185 27715475

[B71] Pope Z. Gao Y. Bolter N. Pritchard M. (2015). Validity and reliability of eating disorder assessments used with athletes: a review. J. Sport Health Sci. 4, 211–221. 10.1016/j.jshs.2014.05.001

[B72] Prather H. Hunt D. McKeon K. Simpson S. Meyer E. B. Yemm T.. (2016). Are elite female soccer athletes at risk for disordered eating attitudes, menstrual dysfunction, and stress fractures? PM R 8, 208–213. 10.1016/j.pmrj.2015.07.003 26188245 PMC8301748

[B73] Probst M. Pieters G. Vanderlinden J. (2008). Evaluation of body experience questionnaires in eating disorders in female patients (AN/BN) and nonclinical participants. Int. J. Eating Disord. 41, 657–665. 10.1002/eat.20531 18446834

[B74] Probst M. Pieters G. Vanderlinden J. (2009). Body experience assessment in non-clinical male and female subjects. Eating Weight Disord. Stud. Anorexia Bulimia Obesity 14, e16–e21. 10.1007/BF03354623 19367132

[B75] Qian J. Wu Y. Liu F. Zhu Y. Jin H. Zhang H.. (2021). An update on the prevalence of eating disorders in the general population: a systematic review and meta-analysis. Eating Weight Disord. Stud. Anorexia Bulimia Obesity. [Epub ahead of print]. 10.1007/s40519-021-01162-z 33834377 PMC8933366

[B76] Ramírez M. Godoy-Izquierdo D. Navarrón E. Jiménez-Torres M. Godoy J. (2018). Estrategias para el manejo del cuerpo en adultos jóvenes: interacción entre las percepciones corporales, la edad y el sexo. Behav. Psychol. 26, 337–357.

[B77] Ramírez M. Godoy-Izquierdo D. Vázquez M. Lara R. Navarrón E. Vélez M.. (2015). Imagen corporal y satisfacción corporal en adultos: Diferencias por sexo y edad. Revista Iberoamericana de Psicología del Ejercicio y el Deporte 10, 63–68.

[B78] Randell R. K. Clifford T. Drust B. Moss S. L. Unnithan V. B. De Ste Croix M. B. A.. (2021). Physiological characteristics of female soccer players and health and performance considerations: a narrative review. Sports Med. 51, 1377–1399. 10.1007/s40279-021-01458-1 33844195 PMC8222040

[B79] Ranganathan P. Pramesh C. S. Aggarwal R. (2017). Common pitfalls in statistical analysis: logistic regression. Perspect. Clin. Res. 8:148. 10.4103/picr.PICR_123_17 28828311 PMC5543767

[B80] Rivas T. Bersab,é R. Jiménez M. Berrocal C. (2010). The eating attitudes test (EAT-26): reliability and validity in spanish female samples. Spanish J. Psychol. 13, 1044–1056. 10.1017/S1138741600002687 20977051

[B81] Rodríguez A. M. Salar N. V. Carretero C. M. Gimeno E. C. Collado E. R. (2015). Eating disorders and diet management in contact sports; EAT-26 questionnaire does not seem appropriate to evaluate eating disorders in sports. Nutrición Hospitalaria 32, 1708–1714. 10.3305/nh.2015.32.4.9214 26545540

[B82] Sabiston C. M. Pila E. Vani M. Thogersen-Ntoumani C. (2019). Body image, physical activity, and sport: a scoping review. Psychol. Sport Exerc. 42, 48–57. 10.1016/j.psychsport.2018.12.010

[B83] Sanders K. (2020). Sportscapes: contested bodies, gender and desire within a female Australian rules football team. Int. Rev. Sociol. Sport 55, 685–702. 10.1177/1012690219837898

[B84] Sandgren S. S. Haycraft E. Plateau C. R. (2020). Nature and efficacy of interventions addressing eating psychopathology in athletes: a systematic review of randomised and nonrandomised trials. Eur. Eating Disord. Rev. 28, 105–121. 10.1002/erv.2704 31916367

[B85] Santos D. A. Silva A. M. Matias C. N. Magalhães J. P. Minderico C. S. Thomas D. M.. (2015). Utility of novel body indices in predicting fat mass in elite athletes. Nutrition 31, 948–954. 10.1016/j.nut.2015.02.003 26059366

[B86] Smolak L. Murnen S. Ruble A. (2000). Female athletes and eating problems: a meta-analysis. Int. J. Eating Disord. 272, 371–380. 10.1002/(SICI)1098-108X(200005)27:4<371::AID-EAT1>3.0.CO;2-Y 10744843

[B87] Soulliard Z. A. Kauffman A. A. Fitterman-Harris H. F. Perry J. E. Ross M. J. (2019). Examining positive body image, sport confidence, flow state, and subjective performance among student athletes and non-athletes. Body Image 28, 93–100. 10.1016/j.bodyim.2018.12.009 30623802

[B88] Steinfeld B. Hartmann A. S. Waldorf M. Vocks S. (2020). Development and initial psychometric evaluation of the body image matrix of thinness and muscularity – female bodies. J. Eating Disord. 8:75. 10.1186/s40337-020-00345-w 33292543 PMC7709434

[B89] Stoyel H. Delderfield R. Shanmuganathan-Felton V. Stoyel A. Serpell L. (2021). A qualitative exploration of sport and social pressures on elite athletes in relation to disordered eating. Front. Psychol. 12:633490. 10.3389/fpsyg.2021.633490 33967900 PMC8103200

[B90] Stoyel H. Slee A. Meyer C. Serpell L. (2020). Systematic review of risk factors for eating psychopathology in athletes: a critique of an etiological model. Eur. Eating Disord. Rev. 28, 3–25. 10.1002/erv.2711 31793151

[B91] Sundgot-Borgen J. Meyer N. L. Lohman T. G. Ackland T. R. Maughan R. J. Stewart A. D.. (2013). How to minimise the health risks to athletes who compete in weight-sensitive sports. Review and position statement on behalf of the ad hoc research working group on body composition, health and performance, under the auspices of the IOC medical commission. Br. J. Sports Med. 47, 1012–1022. 10.1136/bjsports-2013-092966 24115480

[B92] Swami V. García A. A. Barron D. (2017). Factor structure and psychometric properties of a Spanish translation of the Body Appreciation Scale-2 (BAS-2). Body Image 22, 13–17. 10.1016/j.bodyim.2017.05.002 28551528

[B93] Teixidor-Batlle C. Andrés A. Vall-Llovera C. V. (2019). Factores de riesgo de trastornos de la conducta alimentaria asociados a deportes estéticos en deportistas españolas. Revista Española de Educación Física y Deportes 426, 430–437.

[B94] Teixidor-Batlle C. Ventura C. Andrés A. (2021). Eating disorder symptoms in elite Spanish athletes: prevalence and sport-specific weight pressures. Front. Psychol. 11:559832. 10.3389/fpsyg.2020.559832 33574780 PMC7870466

[B95] Thomas D. Erdman K. Burke L. (2016). American College of Sport Medicine Joint Position Statement. Nutrition and athletic performance. Med. Sci. Sports Exercise 48, 543–568. 10.1249/MSS.0000000000000852 26891166

[B96] Thompson J. K. (2004). The (mis)measurement of body image: ten strategies to improve assessment for applied and research purposes. Body Image 1, 7–14. 10.1016/S1740-1445(03)00004-4 18089137

[B97] Thompson J. K. Heinberg L. J. Altabe M. Tantleff-Dunn S. (1999). Exacting Beauty: Theory, Assessment, and Treatment of Body Image Disturbance. Washington DC: American Psychological Association.

[B98] Tiggemann M. (2015). Considerations of positive body image across various social identities and special populations. Body Image 14, 168–176. 10.1016/j.bodyim.2015.03.002 25865460

[B99] Trakman G.ina L. Forsyth A. Devlin B. L. Belski R. (2016). A systematic review of athletes' and coaches' nutrition knowledge and reflections on the quality of current nutrition knowledge measures. Nutrients 8:570. 10.3390/nu8090570 27649242 PMC5037555

[B100] Tylka T. L. (2018). Overview of the field of positive body image, in Body Positive: Understanding and Improving Body Image in Science and Practice, eds Daniels E. A. Gillen M. M. Markey C. H. (Cambridge, UK: Cambridge University Press), 6–33.

[B101] Tylka T. L. Homan K. J. (2015). Exercise motives and positive body image in physically active college women and men: Exploring an expanded acceptance model of intuitive eating. Body Image 15, 90–97. 10.1016/j.bodyim.2015.07.003 26281958

[B102] Tylka T. L. Wood-Barcalow N. L. (2015a). What is and what is not positive body image? Conceptual foundations and construct definition. Body Image 14, 118–129. 10.1016/j.bodyim.2015.04.001 25921657

[B103] Tylka T. L. Wood-Barcalow N. L. (2015b). The body appreciation scale-2: item refinement and psychometric evaluation. Body Image 12, 53–67. 10.1016/j.bodyim.2014.09.006 25462882

[B104] Varnes J. R. Stellefson M. L. Janelle C. M. Dorman S. M. Dodd V. Miller M. D. (2013). A systematic review of studies comparing body image concerns among female college athletes and non-athletes, 1997–2012. Body Image 10, 421–432. 10.1016/j.bodyim.2013.06.001 23856303

[B105] Varnes J. R. Stellefson M. L. Miller M. D. Janelle C. M. Dodd V. Pigg R. M. (2015). Body esteem and self-objectification among collegiate female athletes: does societal objectification make a difference? Psychol. Women Q. 39, 95–108. 10.1177/0361684314531097

[B106] Voelker D. K. Galli N. (2019). Eating disorders in competitive sport and dance, in APA Handbooks in Psychology Series. APA Handbook of Sport and Exercise Psychology, Vol.1. Sport Psychology, eds Anshel M. H. Petrie T.A. Steinfeldt J.A. (Washington DC: American Psychological Association), 585–599.

[B107] Voelker D. K. Petrie T. A. Fairhurst K. Casanave K. (2021). “My body loves me, so I should love it back”: a qualitative evaluation of the bodies in motion program with female collegiate athletes. Sport Exercise Perform. Psychol. 10, 43–58. 10.1037/spy0000211

[B108] Voelker D. K. Petrie T. A. Huang Q. Chandran A. (2019). Bodies in motion: an empirical evaluation of a program to support positive body image in female collegiate athletes. Body Image 28, 149–158. 10.1016/j.bodyim.2019.01.008 30716557

[B109] Voelker D. K. Reel J. J. (2018). Researching eating disorders and body image in sport: challenges and recommendations. J. Clin. Sport Psychol. 12, 473–479. 10.1123/jcsp.2018-0017

[B110] Webb J. B. Wood-Barcalow N. L. Tylka T. L. (2015). Assessing positive body image: contemporary approaches and future directions. Body Image 14, 130–145. 10.1016/j.bodyim.2015.03.010 25910972

[B111] Wilinski W. (2012). Gender identity in female football players. Hum. Mov. 13, 40–47. 10.2478/v10038-012-0003-8

[B112] Wilson P. B. Madrigal L. A. Burnfield J. M. (2016). Weight control practices of Division I National Collegiate Athletic Association athletes. Phys. Sportsmed. 44, 170–176. 10.1080/00913847.2016.1149420 26831597

[B113] Wood-Barcalow N. L. Augustus-Horvath C. L. (2018). Clinical applications of positive body image, in Body positive: understanding and improving body image in science and practice, eds Daniels E. A. Gillen M. M. Markey C. H. (Cambridge, UK: Cambridge University Press), 235–261.

[B114] Wood-Barcalow N. L. Tylka T. L. Augustus-Horvath C. L. (2010). “But I like my body”: positive body image characteristics and a holistic model for young-adult women. Body Image 7, 106–116. 10.1016/j.bodyim.2010.01.001 20153990

